# Evaluating the Solvent and pH Effect in the Inversion of the Circular Dichroism Signal of *S*‐Naproxen

**DOI:** 10.1002/chir.70137

**Published:** 2026-08-19

**Authors:** Lucas Chuman Santana, Eduardo de Faria Franca, Valdecir Farias Ximenes, Aguinaldo Robinson de Souza, Nelson Henrique Morgon

**Affiliations:** ^1^ Department of Physical Chemistry, Institute of Chemistry University of Campinas (UNICAMP) Campinas SP Brazil; ^2^ Laboratory of Crystallography and Computational Chemistry, Chemistry Institute Federal University of Uberlândia – UFU Uberlândia MG Brazil; ^3^ Department of Chemistry, School of Science São Paulo State University (UNESP) Bauru São Paulo Brazil

**Keywords:** electronic circular dichroism, molecular dynamics, *S*‐naproxen, solvent effect, TD‐DFT

## Abstract

*S*‐Naproxen (NPX) exhibits an unusual inversion of its ECD spectrum upon solvent change, a phenomenon that this work analyzes both experimentally and theoretically. By evaluating NPX in water, ethanol, and acetonitrile, it was observed that pH variation shifts the chemical equilibrium between protonated and deprotonated forms, making this transition the direct cause of the ECD signal inversion. Molecular dynamics and TD‐DFT (CAM‐B3LYP/aug‐cc‐pVDZ) calculations corroborated the experimental data, demonstrating that structural changes alter the predominant electronic transitions from π → π* in NPX to n → π* in the NPX^−^ anion, as evidenced by transition density differences and natural transition orbitals (NTOs). These findings demonstrate that explicit solvent treatment and statistical analysis are requisite to accurately reproduce the data, validating that the negative signal in water and the positive signal in acetonitrile is governed by the predominant species dictated by the chemical equilibrium.

## Graphical abstract

This study provides a theoretical and experimental analysis of the electronic circular dichroism (ECD) spectrum inversion observed in *S*‐naproxen when deprotonated through pH control.

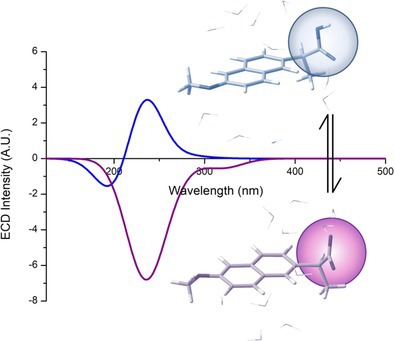

## 1 Introduction

*S*‐naproxen (NPX) is a chiral nonsteroidal anti‐inflammatory drug (NSAID) that exhibits solvent‐dependent electronic circular dichroism (ECD) behavior [1]. The first ab initio calculations of optical rotatory dispersion (ORD) and absolute configuration started by Polavarapu [2] who provided the foundation for modern computational studies. Today, well‐established protocols enable the analysis of ECD [3, 4, 5, 6, 7] and ORD correlations via the Kramers–Kronig transform [8], offering deeper insights into chiral phenomena. However, a fundamental challenge in accurately simulating ECD spectra lies in incorporating solvent effects, as they profoundly influence experimental outcomes. This has been demonstrated through ORD studies of propylene oxide in various solvents, where the rigid nature of the molecule ensures that observed changes in optical activity are attributable solely to solvent interactions [9]. Consequently, the relationship between optical activity and factors such as (i) concentration dependence, (ii) solvent nature, (iii) solvent purity, (iv) temperature, and (v) molecular self‐association is well established [10].

The impact of solvent effects extends beyond fundamental studies to practical applications, particularly in chiral separations. A striking example is the resolution of (*R*, *S*)‐Naproxen enantiomers, which relies on solvents such as methanol and acetonitrile [8]. When combined with high‐performance liquid chromatography (HPLC) coupled with a circular dichroism detector, these solvents offer distinct advantages, as the technique selectively detects chiral compounds, surpassing traditional screening methods in pharmaceutical analysis [11].

A notable manifestation of solvent effects in ECD spectroscopy occurs in (*S*)‐naproxen (NPX), which exhibits spectral signal inversion in acetonitrile relative to ethanol or water. This unusual behavior has been widely reported in the literature [1]. Theoretical approaches have rationalized this phenomenon using implicit solvent models, a well‐established methodology [12, 13, 14, 15]. However, this methodology has not produced strong correlations between theoretical and experimental spectral profiles. A significant breakthrough in modeling solvent effects was achieved by Chiara Cappelli [16], who demonstrated that explicit solvent modeling, coupled with molecular dynamics (MD), provides a more accurate representation of the experimental ECD spectrum of NPX in water.

The present work investigates the inversion of the ECD signal of NPX as a function of solvent and pH using experimental measurements and theoretical calculations. Explicit solvent effects were considered.

## 2 Methodology

The computational ECD spectra were calculated according to the protocols of Polavarapu and Cappelli [6, 16]. Conformational ensembles were obtained via MD simulations, followed by cluster‐based population analysis in GROMACS. Subsequent quantum chemistry calculations were executed using the TD‐DFT method.

### 2.1 MD Conformational Studies

**FIGURE 1:**
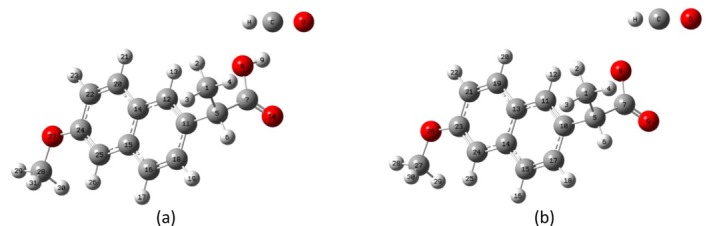
Representation of labels in sequence describes the force field of NPX (a) and deprotonated NPX^−^ (b).

We initially performed MD simulations using the structure of NPX from PubChem CID 156391 [17]. For the simulation of NPX^−^ (deprotonated NPX), the carboxylate form was obtained by removing the acidic proton from the carboxyl group. For the MD simulations, it was necessary to develop a force field, following these steps: (i) Atomic charges were determined using the restrained electrostatic potential (RESP) method [18, 19] implemented in the NWChem 6.6 program [20]. (ii) The Ryckaert–Bellemans potential, which describes the torsional dihedrals in the force field, was taken from the literature [21]. (iii) The remaining parameters were taken from the OPLS‐AA [22] force field available in the GROMACS library. Tables S1–S4 present the PDB and force field parameters of NPX and NPX^−^. Figure 1 shows the structures of NPX and NPX^−^ with their respective atoms labeled as defined in the developed force field.

MD simulations were executed using GROMACS 2020.1 [23]. Each system comprised one solute molecule solvated with exactly 1000 solvent molecules in a cubic cell under periodic boundary conditions. First, energy minimization was achieved via the steepest descent method [24] until the maximum force fell below 1.0 kJ mol^−1^ nm^−1^. Subsequently, a 500‐ps NVT MD simulation was carried out at 300 K, constraining hydrogen bond lengths with the LINCS algorithm [25]. Electrostatic interactions were computed using Particle‐Mesh Ewald (PME) summation [26]. Equilibration continued in the NPT ensemble for another 500 ps at 1 bar, maintained by the Parrinello–Rahman barostat [27, 28]. The production run lasted 1000 ps, with configurations sampled every 10 ps. Finally, structures were clustered using the GROMOS method [29] with a 0.05 nm cutoff, defining representatives based on the minimum average RMSD within each cluster. Regarding MD, the images were created using the Visual Molecular Dynamics (VMD) software [30] and the PyMOL software [31].

### 2.2 TD‐DFT Electronic Circular Dichroism Computations

All the quantum mechanical calculations were performed using the Gaussian 16 software package [32], with all figures associated with Gaussian 16 generated via Gaussview 5.0 [33]. The quantum mechanical analyses began with the clustering process from the Gromacs software package. The theoretical ECD spectra for each structure were obtained using time‐dependent density functional theory (TD‐DFT) [34], considering the first 15 excited states computed at the CAM‐B3LYP functional [35] with the aug‐cc‐pVDZ basis set. Natural transition orbitals (NTOs), ground‐state electron density, and excited‐state electron density were analyzed to describe the nature of each electronic transition.

### 2.3 Experimental ECD in Function of pH

The experimental ECD measurements followed the protocol described by Ximenes [1]. The (*S*)‐naproxen enantiomer was acquired from Sigma‐Aldrich Chemical Co. (St. Louis, Missouri), and its stock solution (10 mM) was prepared in ethanol. The solvents ethanol and acetonitrile used were of analytical grade. The ECD spectra were obtained with a resolution of 1 nm, a response time of 1 s, and a scanning speed of 50 nm/min. The spectra of (*S*)‐naproxen were recorded at a concentration of 2 μM, in water, ethanol, and acetonitrile, in the range of 200 to 300 nm, at 25°C. The measurements were performed on a Jasco J‐815 spectropolarimeter (Jasco, Japan), equipped with a thermostatically controlled cell holder. A 3‐mL quartz cuvette with an optical path length of 10 mm was used in all analyses. The baseline spectrum (solvent) was subtracted from all measurements.

The first NPX measurements were performed in pure water. Subsequently, pH was controlled using acetate and MES (2‐(N‐morpholino)ethanesulfonic acid) buffers. The pH‐controlled measurements were performed at pH 3.0, 3.7, 4.5, 5.5, and 6.5. The MES buffer was used for pH 5.5 and 6.5, and the acetate buffer was used for the other values.

Subsequently, two NPX measurements were performed in acetonitrile and ethanol (pure solvents). In addition, two pH extrapolation measurements were performed, in which the target pH was imposed by the addition of reagents without buffer control. For NPX in ethanol, 1.0 M hydrochloric acid was added to establish an acidic medium (ETH + Ac.); for NPX in acetonitrile, 8.0 M sodium hydroxide was used to obtain a basic medium (ACN + NaOH).

## 3 Results and Discussion

### 3.1 Experimental ECD of NPX–pH Effect

**FIGURE 2:**
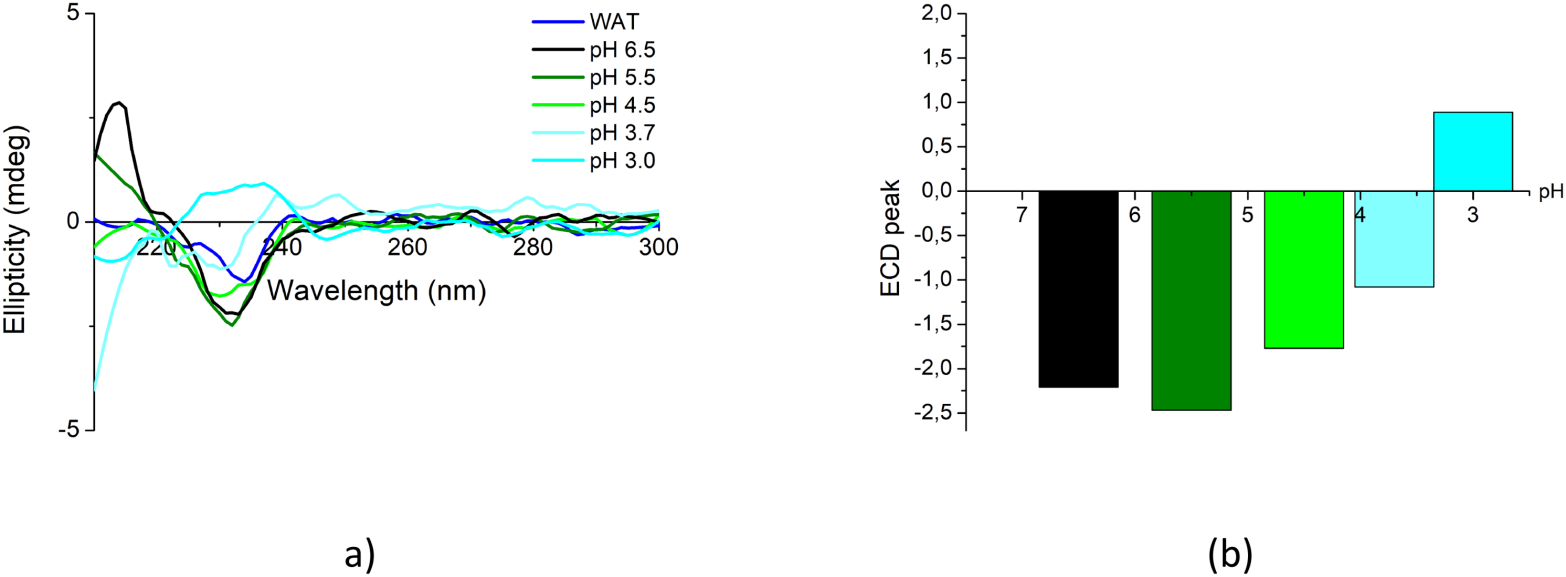
Experimental ECD spectra of NPX in WAT in pure solvent and with pH control (a) and the variation of the ECD peak values at different pH levels (b) highlight the signal inversion at pH 3.0.

The pH exerts a profound influence on the ECD spectrum of NPX. Figure 2 presents the ECD spectra of NPX in WAT, illustrating the effect of pH control in this solvent. NPX exhibits deprotonation equilibrium due to its carboxylic acid group. Consequently, at high pH values, the equilibrium shifts toward the formation of the deprotonated species, NPX^−^. Conversely, at low pH values, the increased concentration of hydronium ions drives the equilibrium toward the protonated NPX form. In water, the pKa of NPX is 4.2 [36, 37]; experimentally, an inversion of the ECD signal is observed at pH 3.0.

Considering the proton‐accepting ability of the solvent, ethanol exhibits a behavior similar to that of water. In pure ethanol, NPX presents a negative peak (as observed in water), indicating that the presence of the deprotonated form (NPX^−^) significantly influences the spectrum. Upon the addition of acid to the medium, the equilibrium shifts toward the formation of protonated NPX, and, similar to the aqueous system, the spectral profile becomes positive. Thus, the presence of NPX^−^ in these pure solvents dictates the ECD spectrum, yielding a negative curve profile; however, forcing the equilibrium to predominantly produce protonated NPX shifts the curve to a positive profile.

**FIGURE 3:**
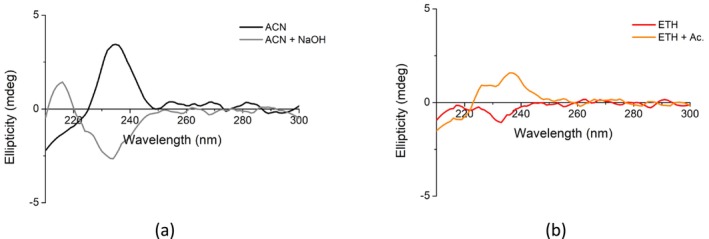
Experimental ECD spectra of NPX in ACN (a) and ETH (b) in pure solvent and with pH control.

Furthermore, in ACN, there is practically no deprotonation of NPX, which imparts a distinct spectral profile in the pure solvent. This behavior is supported by literature values for the pKa of carboxylic acids; for instance, the pKa of acetic acid is 4.76 in water, whereas it increases drastically to 23.51 in ACN [38]. In this context, forcing the deprotonation of NPX to NPX^−^ in ACN requires the addition of NaOH to the system. Upon generating NPX^−^ through this addition, the ECD spectral profile is altered once again, confirming that the presence of the deprotonated species (NPX^−^) is the fundamental factor dictating the resulting ECD spectrum. Figure 3 illustrates the behavior of the ECD spectra for both the protonated (NPX) and deprotonated (NPX^−^) forms in ethanol and ACN.

### 3.2 MD Conformational Studies

**FIGURE 4:**
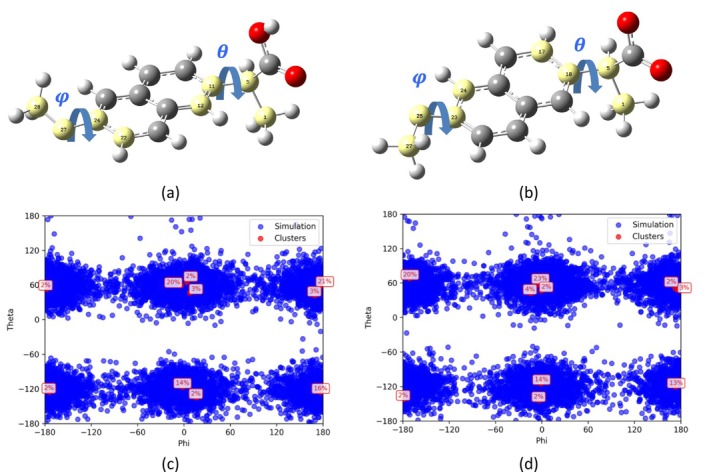
Structures of NPX (a) and NPX^−^ (b) with the identification of the dihedral atoms. Conformations extracted from molecular dynamics in WAT for NPX (c) and NPX^−^ (d).

The MD of NPX and NPX^−^ generates a large number of conformers, with one selected and represented by clustering tools of Gromacs software. The cluster analysis of NPX in water is similar to the literature [16, 39]. Each simulation considered many clusters representing a minimum of 90%. The average density in ACN (725.823 kg.m^−3^), WAT (992.87 kg.m^−3^), and ETH (788.851 kg.m^−3^) is similar to experimental data [40, 41, 42]. Figure 4 shows the dihedrals involved in conforming identification and all the conformations saved in the dynamics, with emphasis on the cluster used in further calculations. The corresponding clusters for NPX in ETH and ACN are shown in Figure S1.

### 3.3 TD‐DFT Electronic Circular Dichroism Computations

#### 3.3.1 ECD of NPX and NPX^−^ in Gas Phase

Theoretical ECD spectra were evaluated using different basis sets and functionals. The results for NPX with only one water molecule are available in Figure S2. The ECD spectrum profile of all theoretical methods is similar to the literature [16]; consequently, all the results are presented using the same methodology as reported in the literature (CAM‐B3LYP/aug‐cc‐pVDZ).

**FIGURE 5:**
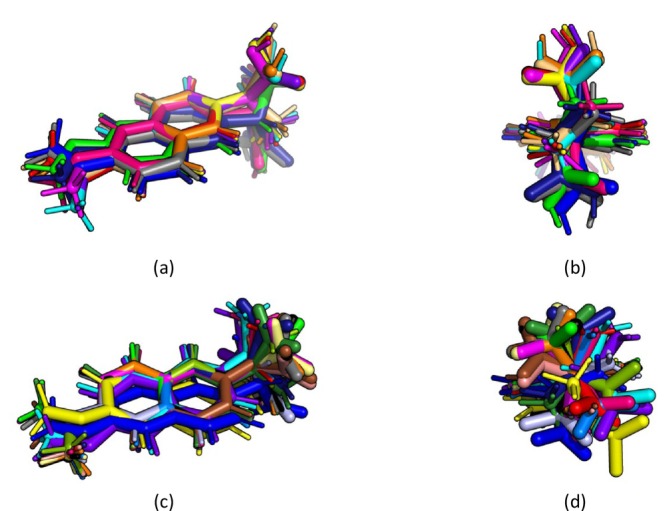
NXP Clusters in isometric (a) and front (b) view. NPX^−^ clusters in the isometric (c) and front view (d).

For the gas phase simulation, 12 clusters were computed to represent 90% of NPX dynamics and 19 clusters for the NPX^−^ dynamic. All cluster structures for NPX and NPX^−^ are shown in Figure 5.

**FIGURE 6:**
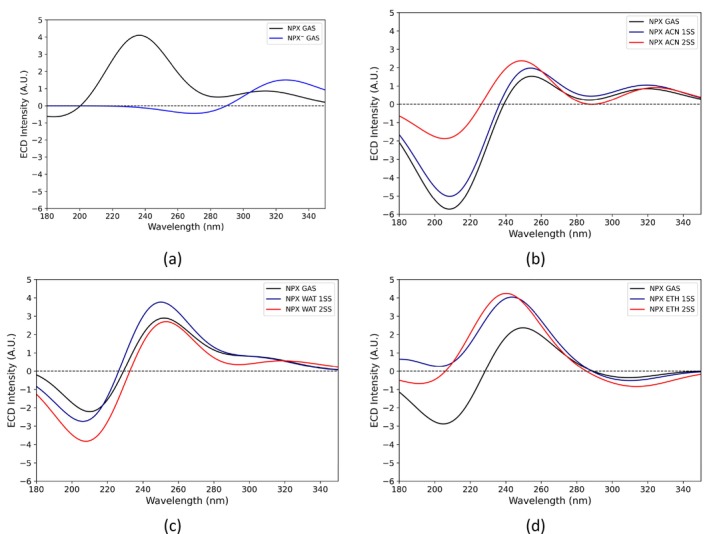
Theoretical ECD spectra of NPX and NPX^−^ in the gas phase (a). NPX in ACN (b), WAT (c), and ETH (d)—computed the cluster isolated from solvent (gas phase), the first and second solvation shells. The CAM‐B3LYP/aug‐cc‐pVDZ/TD (NStates = 15) level was used.

The theoretical ECD spectrum for each cluster is provided in Figure S3. By applying the percentage contribution of each cluster, according to their respective population's weights, the final average ECD spectrum was obtained and is presented in Figure 6. In this case, accounting for the statistical contribution of the clusters is crucial for the resultant spectrum. Notably, there is no inversion in the ECD signals; instead, the NPX^−^ spectrum exhibits a wavelength shift.

#### 3.3.2 ECD of NPX and NPX^−^ in Solvent

When the NPX dynamics include ACN, WAT, and ETH as solvents (Figure 6), the resulting ECD signals are similar in all three. This differs from the experimental data of this work and the literature [1], which reports that the ACN spectrum is opposite to those of WAT and ETH. Furthermore, no significant differences were observed between the first and second solvation shells (a comparison using the continuum solvation model is shown in Figure S4). The indicated “gas phase” calculations correspond to structures where the solvent molecules from the dynamics were removed before the ECD calculation, while maintaining the NPX geometry (which also did not show significant changes in the ECD spectrum). Consequently, because the three spectral profiles are identical, merely including the solvent effect was not sufficient to reproduce the experimental spectrum, which aligns with the literature.

**FIGURE 7:**
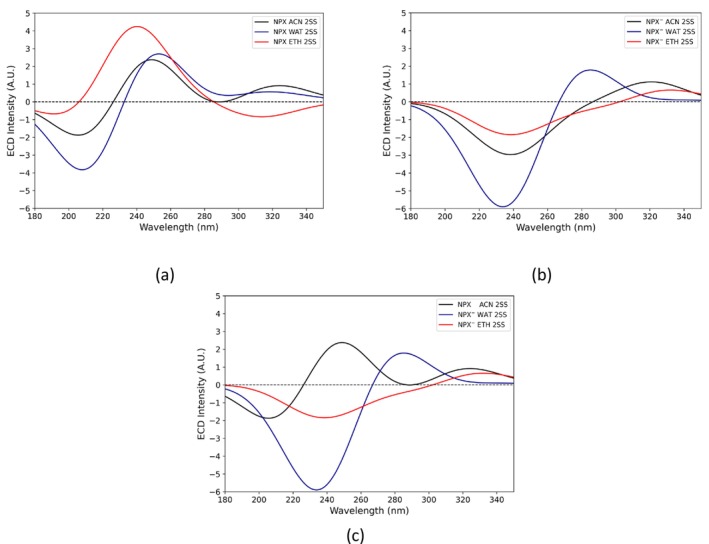
Theoretical ECD spectra of NPX (a), NPX^−^ (b), and the combination of these two structures, NPX/NPX^−^ (c), in the solvents studied (ACN, WAT, and ETH). The CAM‐B3LYP/aug‐cc‐pVDZ/TD (NStates = 15) level was used.

With the simulation of NPX^−^, the inversion of the ECD spectrum occurs in all solvents. Thus, the rationale for describing the inversion of the ECD spectrum as the solvent changes agrees with the experimental data of this work and the literature [1], which is the deprotonation of NPX—by changing the major structure as a function of pH, a new ECD spectrum is obtained. Figure 7 compares the spectra of NPX and NPX^−^ in the studied solvents, and the combination of NPX for ACN and NPX^−^ for the WAT and ETH solvents describes the unusual solvent effect that causes the inversion, faithfully representing the literature [1], which was also identified experimentally in this work.

**FIGURE 8:**
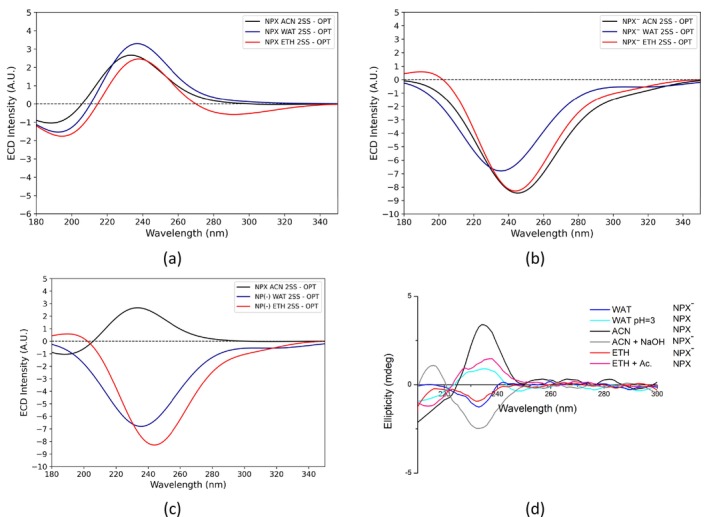
Theoretical ECD spectra of optimized clusters of NPX (a), NPX^−^ (b), and the combination of these two structures, NPX/NPX^−^, in (c) for the solvents studied (ACN, WAT, and ETH). Experimental ECD spectra of NPX in pure solvent and under pH control are shown for comparison (d). The CAM‐B3LYP/aug‐cc‐pVDZ/TD (NStates = 15) level of theory was used.

For future analyses of the cluster structures, the solvent was kept frozen, and the solute optimization was performed (OPT). Figure 8 presents the theoretical ECD spectrum in the solvents studied with the optimized structures of the NPX and NPX^−^ clusters. It is observed that for NPX, the exclusion of the solvent after optimization (gas phase) did not cause a significant change in the ECD spectrum profile. For NPX^−^, however, the gas phase showed a different behavior compared with when the spectrum was simulated with the second solvation shell of the solvent. Consequently, the correlation between NPX/NPX^−^ with the second solvation shell is performed (also in Figure 8), reproducing the experimental data with pH control and with a spectrum profile extremely similar to the literature [1]. Thus, the treatment of the solvent effect was necessary to correctly describe the experimental ECD spectrum; even with the deprotonation of NPX, without the explicit treatment of the solvent, the ECD spectrum profile would not resemble the experimental one as much as with the gas phase. The deprotonation of NPX was crucial for describing the inversion of the ECD spectrum, justifying its alteration simply by the change of the system from NPX to NPX^−^, with the pH governing the unusual behavior of the NPX ECD spectrum. This same spectral profile for the NPX^−^ anion clusters was evaluated at the CAM‐B3LYP/aug‐cc‐pVDZ/TD (NStates = 30) level of theory. The addition of more states did not modify the spectral profile, as the intensity of the added peaks is low, as seen in Figure S5.

#### 3.3.3 Dipole Moment of NPX and NPX^−^

**FIGURE 9:**
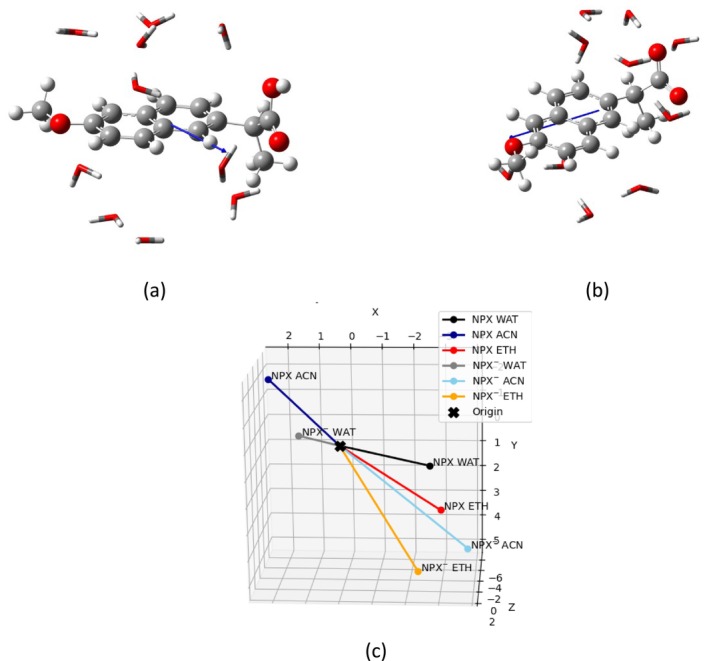
Resulting dipole moments of the NPX (a) and NPX^−^ (b) systems in WAT, and the resulting vectors for both species in all evaluated solvents (c).

The dipole moment is considered an important parameter for evaluating ECD spectrum inversion [43]. Upon evaluating the deprotonation of NPX in WAT and ACN, the resulting dipole moment exhibits a reversal of direction, as reported in the literature [43]. However, the angle between the vectors is not exactly 180°, and for the case of NPX and NPX^−^ in ethanol, this pattern of dipole moment inversion was not observed. Figure 9 presents the structures of NPX and NPX^−^ in WAT with their respective dipole moments, as well as the representation of the dipole moment for this system in ACN and ETH. Figure S6 presents the structures of NPX and NPX^−^ in ACN and ETH.

#### 3.3.4 Electron Density Difference (EDD) and NTO of NPX and NPX^−^

**FIGURE 10:**
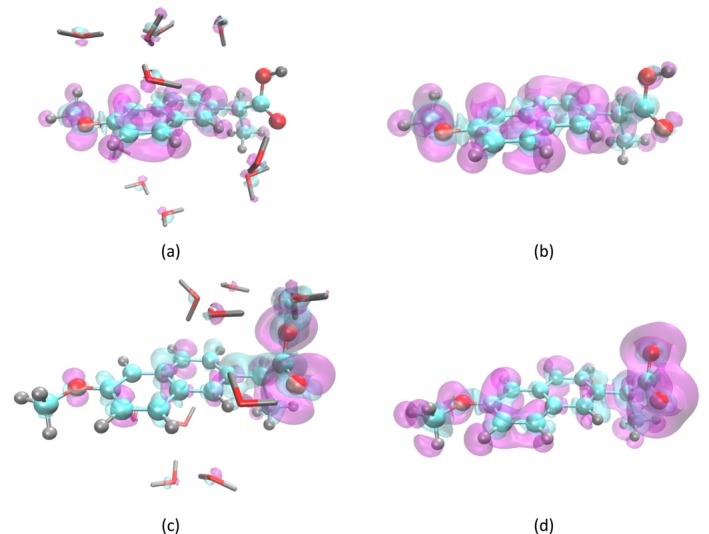
Density transition difference Iso‐surface of NPX/WAT (a), NPX/Gas (b), NPX(^−^)/WAT (c), and NPX^−^/Gas (d) via EDD analysis (Iso‐values = 0.001).

The transition density is a quantity that evaluates the movement of charges during the electronic transition, serving as a visual representation that partially explains the behavior of the ECD [44]. This occurs because, according to the literature, the ECD is governed by the product of the electric and magnetic dipole transition moments [3, 45]. In Figure 10, the movement between the initial and final states is represented, with the turquoise color representing the origin (loss) of electron density and the purple color representing the final position (gain) of electron density [46]. In this context, it is observed that between the system in the gas phase and that with the inclusion of the solvent, the density difference also encompasses the solvent, indicating the participation of the solvent in the ECD spectrum. Furthermore, between the NPX^−^ and NPX systems, there is greater participation of the carboxylic acid group in NPX^−^, whereas the NPX system exhibits greater distribution over the aromatic ring. Figures S7–S12 show the correlation between NPX and NPX^−^ in ETH and ACN.

The electron density deformation capacity is evaluated by the polarizability (Table 1), the increase of which is significant upon the addition of solvent compared with the gas phase. Regarding the protonated and deprotonated systems of NPX, there is no significant change in polarizability; however, the deprotonated system, NPX^−^, is slightly more polarizable than NPX in the gas phase. This effect is lost with the addition of explicit solvent, with both systems showing practically the same polarizability behavior and exhibiting no difference between NPX and NPX^−^. Furthermore, with the inclusion of explicit solvent, the polarizability increases in both cases, reaching values between 41 and 42 Å^3^. The hyperpolarizability, on the other hand, does not exhibit a consistent effect in the analyzed clusters, showing sometimes an increase and sometimes a quenching of the nonlinear optical response. A decrease in the nonlinear optical activity was expected in all cases where the explicit solvent effect is included. Similar hyperpolarizability analyses for NPX and NPX^−^ in ACN and ETH are presented in Table S5.

**TABLE 1:** Isotropic polarizability (α) and total first hyperpolarizability ($\beta_{\mathrm{tot}}$) of neutral (NPX) and anionic (NPX^−^) naproxen clusters in gas and solvent phases.

System		Solvent	Polarizability Å^3^	Hyperpolarizability 10^−33^ esu
NPX	Cluster 01	Gas	27.6212	5189.5934
		WAT (2SS)	41.0045	8385.5933
	Cluster 02	Gas	27.7347	4245.2214
		WAT (2SS)	41.3791	4988.8887
	Cluster 03	Gas	27.7507	4456.8085
		WAT (2SS)	41.1076	3527.4594
NPX^−^	Cluster 01	Gas	29.5563	5707.3863
		WAT (2SS)	42.2153	2453.5573
	Cluster 02	Gas	29.1306	4513.5937
		WAT (2SS)	41.9496	4774.9773
	Cluster 03	Gas	29.6397	8245.1610
		WAT (2SS)	42.5997	5123.4220

**FIGURE 11:**
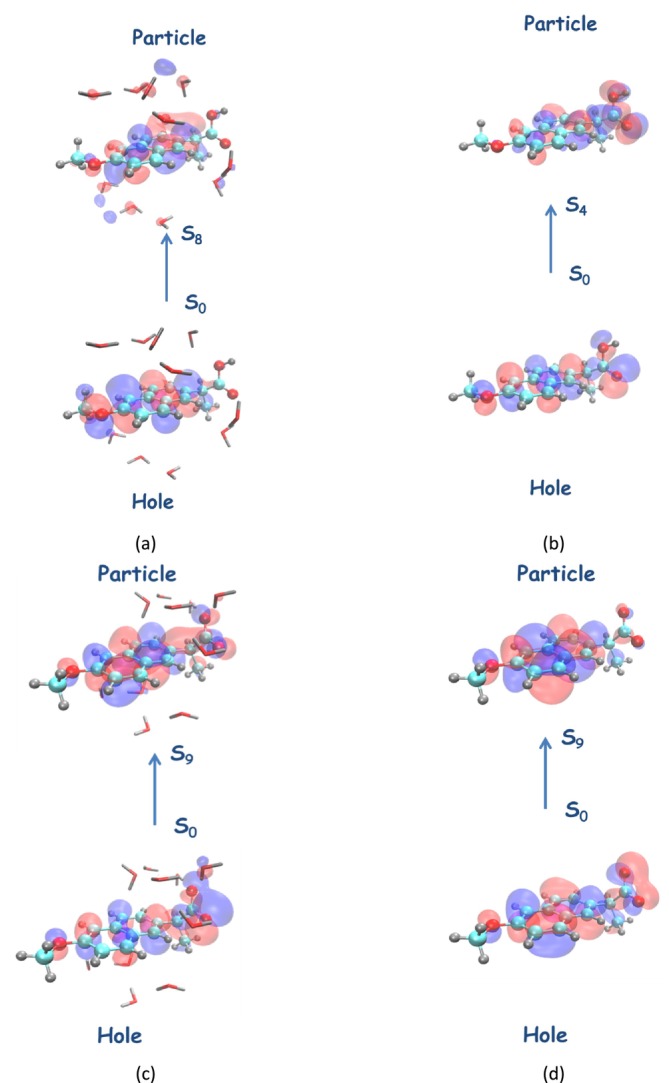
The natural transition orbital (NTO) hole and particle patterns of NPX/WAT (a), NPX/GAS (b), NPX^−^ /WAT (c), and NPX^−^ /GAS (d)—Iso‐values = 0.020000.

The NTOs show the same behavior, namely, the presence of orbitals involving the solvent, indicating the greater participation of the solvent compared with the gas phase, and showing that, upon deprotonation, the acid group, which previously did not participate significantly in the electronic transition, becomes expressive. Therefore, it is determined that the major transitions for NPX are π → π*, which is similar to the description in the literature [47]. NPX^−^, which also exhibits π and π* type orbitals, begins to present n → π* transitions. Figure 11 shows the NTOs to NPX and NPX^−^ dominant cluster in WAT, the structures of the other clusters and solvents are provided in Figure S13–S18.

## 4 Conclusions

The experimental ECD measurement of NPX was performed in three pure solvents (WAT, ACN, and ETH); additionally, the pH was varied in these three solvents, and it was observed that varying the pH causes an inversion in the ECD spectrum, which is justified by the deprotonation of the carboxylic acid group present in NPX, governed by the chemical equilibrium associated with deprotonation. This same inversion effect in the ECD spectrum was evaluated using theoretical chemistry. Several conformations were generated by MD, yielding statistically weighted ECD spectra, as each conformation exhibits a distinct ECD spectrum profile. The deprotonation of NPX also generated an inversion in the ECD spectra, as predicted by theoretical chemistry calculations. Deprotonation changes the transition orbitals of the π → π* type, predominantly present in the NPX structure, to n → π* in the case of NPX^−^. This transition difference is also observed in the transition density difference, which is shifted toward the carboxylic acid group. In addition to deprotonation, it was determined that the explicit solvent also participates in the transition orbitals and is similarly present in the transition density difference; thus, it is concluded that the explicit solvent was essential for the quality of the generated spectra and for the agreement between theoretical and experimental data. The participation of the solvent was also evaluated by the increase in the polarizability of NPX and NPX^−^ compared with the gas phase. In contrast, the hyperpolarizability data did not show consistent behavior in the performed analyses.

## Supporting information


**Table S1:** NPX PDB file.
**Table S2:** NPX− PDB file.
**Table S3:** NPX full force field (.itp file).
**Table S4:** NPX− full force field (.itp file).
**Figure S1:** NXP (a) and NPX− (d) structure with the identification of dihedral atoms. Saved structures conformation of molecular dynamics in NPX in ACN (b), ETH (c), and NPX− in ACN (e) and ETH (f).
**Figure S2:** Evaluation of solvent influence in NPX ECD spectra. Changing the Pople, Dunning, and Karlsruhe bases set in front of DFT functional: B3LYP (a), CAM‐B3LYP (b), M06‐2X (c), and ωB97XD (d). All analysis were performed in TD (NStates = 15).
**Figure S3:** All individual ECD spectra to each cluster of NPX GAS (a), WAT (b), ACN (c), and ETH (d) and to the NPX− GAS (e), WAT(f), ACN (g), and ETH (h). The CAM‐B3LYP/aug‐cc‐pVDZ/TD (NStates = 15) level was used.
**Figure S4:** Theoretical ECD spectra of NPX and NPX− in the gas phase (a). NPX in ACN (b), WAT (c), and ETH (d) computed the cluster isolated from solvent (gas phase), the first and second solvation shells. The CAM‐B3LYP/aug‐cc‐pVDZ/TD (NStates = 15) level was used.
**Figure S5:** Comparison of the ECD spectra with NStates = 15 and NStates = 30 for NPX− in ACN (a), WAT (b), and ETH (c). The CAM‐B3LYP/aug‐cc‐pVDZ/level was used.
**Figure S6:** Resulting dipole moment to the NPX/ACN (a), NPX−/ACN (b), NPX/ETH (c), and NPX−/ETH (d) systems.
**Figure S7:** Density transition difference iso‐surface of NPX via EDD analysis (iso‐values = 0.001). Cluster 1: ACN (a)–GAS (b). Cluster 2: ACN (c)–GAS (d). Cluster 3: ACN (e)–GAS (f).
**Figure S8:** Density transition difference iso‐surface of NPX via EDD analysis (iso‐values = 0.001). Cluster 1: ETH (a)–GAS (b). Cluster 2: ETH (c)–GAS (d). Cluster 3: ETH (e)–GAS (f).
**Figure S9:** Density transition difference iso‐surface of NPX via EDD analysis (iso‐values = 0.001). Cluster 1: WAT (a)–GAS (b). Cluster 2: WAT (c)–GAS (d). Cluster 3: WAT (e)–GAS (f).
**Figure S10:** Density transition difference iso‐surface of NPX− via EDD analysis (iso‐values = 0.001). Cluster 1: ACN (a)–GAS (b). Cluster 2: ACN (c)–GAS (d). Cluster 3: ACN (e)–GAS (f).
**Figure S11:** Density transition difference iso‐surface of NPX− via EDD analysis (iso‐values = 0.001). Cluster 1: ETH (a)–GAS (b). Cluster 2: ETH (c)–GAS (d). Cluster 3: ETH (e)–GAS (f).
**Figure S12:** Density transition difference iso‐surface of NPX− via EDD analysis (iso‐values = 0.001). Cluster 1: WAT (a)–GAS (b). Cluster 2: WAT (c)–GAS (d). Cluster 3: WAT (e)–GAS (f),
**Table S5:** Isotropic polarizability (α) and total first hyperpolarizability (βtot) of neutral (NPX) and anionic (NPX−) naproxen clusters in gas and solvent phases.
**Figure S13:** NPX NTO Orbitals. Cluster 1: Particle ACN (a)–GAS (b), Hole ACN (c)–GAS (d). Cluster 2: Particle ACN (e)–GAS (f), Hole ACN (g)–GAS (h). Cluster 3: Particle ACN (i)–GAS (j), Hole ACN (k)–GAS (l). Iso‐values = 0.020000.
**Figure S14:** NPX NTO Orbitals. Cluster 1: Particle ETH (a)–GAS (b), Hole ETH (c) – GAS (d). Cluster 2: Particle ETH (e) – GAS (f), Hole ETH (g) – GAS (h). Cluster 3: Particle ETH (i)–GAS (j), Hole ETH (k)–GAS (l). Iso‐values = 0.020000.
**Figure S15:** NPX NTO Orbitals. Cluster 1: Particle WAT (a)–GAS (b), Hole WAT (c)–GAS (d). Cluster 2: Particle WAT (e)–GAS (f), Hole WAT (g)–GAS (h). Cluster 3: Particle WAT (i)–GAS (j), Hole WAT (k)–GAS (l). Iso‐values = 0.020000.
**Figure S16:** NPX− NTO Orbitals. Cluster 1: Particle ACN (a)–GAS (b), Hole ACN (c)–GAS (d). Cluster 2: Particle ACN (e)–GAS (f), Hole ACN (g)–GAS (h). Cluster 3: Particle ACN (i)–GAS (j), Hole ACN (k)–GAS (l). Iso‐values = 0.020000.
**Figure S17:** NPX− NTO Orbitals. Cluster 1: Particle ETH (a)–GAS (b), Hole ETH (c)–GAS (d). Cluster 2: Particle ETH (e)–GAS (f), Hole ETH (g)–GAS (h). Cluster 3: Particle ETH (i)–GAS (j), Hole ETH (k)–GAS (l). Iso‐values = 0.020000.
**Figure S18:** NPX− NTO Orbitals. Cluster 1: Particle WAT (a)–GAS (b), Hole WAT (c)–GAS (d). Cluster 2: Particle WAT (e)–GAS (f), Hole WAT (g)–GAS (h). Cluster 3: Particle WAT (i)–GAS (j), Hole WAT (k)–GAS (l). Iso‐values = 0.020000.

## Data Availability

The .PDB and .ITP files of NPX and NPX^−^, necessary to perform the GROMACS dynamics, are openly available in the Unicamp Research Data Repository (DOI: https://doi.org/10.25824/redu/J4MZNE). All the structures used in this study are openly available in the Unicamp Research Data Repository (DOI: https://doi.org/10.25824/redu/ZSYV1G).
