# Supplementary material for: Evaluating the Solvent and pH Effect in the Inversion of the Circular Dichroism Signal of S‐Naproxen
Source: Chirality. 2026 Aug 19;38(9):e70137. doi: 10.1002/chir.70137 (PMC13487506; doi:10.1002/chir.70137)
Supplement: Supplementary file 1 [file CHIR-38-e70137-s001.docx]

**SUPPORTING INFORMATION**

**Evaluating the solvent-and pH effect in the inversion of the circular dichroism signal of S-Naproxen**

Lucas Chuman Santana, * ^[a]^ Eduardo de Faria Franca,^[b]^ Valdecir Farias Ximenes,^[c]^ Aguinaldo Robinson de Souza,^[c]^ Nelson Henrique Morgon ^[a]^

The following tables contain the available non-bonded and bonded parameters for the OPLS-AA force field of NPX/NPX^-^. In sequence all image

**COMPLEMENTARY FIGURES AND TABLES**

Table S1. NPX PDB file.

COMPND	Configuration number : 780L =	39.2084 39.2084 39.2084

AUTHOR	GENERATED BY OPEN BABEL 3.0.0

HETATM	1 C1 NAP	1	-3.587 -0.134	1.803 1.00 0.00	C

HETATM	2 HC1 NAP	1	-3.272 -1.177	1.865 1.00 0.00	H

HETATM	3 HC2 NAP	1	-3.154	0.403	2.649 1.00 0.00	H

HETATM	4 HC3 NAP	1	-4.675 -0.105	1.900 1.00 0.00	H

HETATM	5 C2 NAP	1	-3.130	0.521	0.487 1.00 0.00	C

HETATM	6 H2 NAP	1	-3.479	1.555	0.470 1.00 0.00	H

HETATM	7 C3 NAP	1	-3.783 -0.112 -0.735 1.00 0.00	C

HETATM	8 OH NAP	1	-3.730 -1.469 -0.713 1.00 0.00	O

HETATM	9 H3 NAP	1	-4.135 -1.789 -1.534 1.00 0.00	H

HETATM	10 O3 NAP	1	-4.293	0.493 -1.643 1.00 0.00	O

HETATM	11 C4 NAP	1	-1.611	0.546	0.326 1.00 0.00	C

HETATM	12 C5 NAP	1	-0.851 -0.607	0.331 1.00 0.00	C

HETATM	13 H51 NAP	1	-1.332 -1.576	0.425 1.00 0.00	H

HETATM	14 C6 NAP	1	0.558 -0.566	0.201 1.00 0.00	C

HETATM	15 C7 NAP	1	1.215	0.693	0.059 1.00 0.00	C

HETATM	16 C8 NAP	1	0.417	1.864	0.050 1.00 0.00	C

HETATM	17 H81 NAP	1	0.899	2.832 -0.061 1.00 0.00	H

HETATM	18 C9 NAP	1	-0.950	1.791	0.177 1.00 0.00	C

HETATM	19 H91 NAP	1	-1.539	2.703	0.161 1.00 0.00	H

HETATM	20 C10 NAP	1	1.355 -1.745	0.206 1.00 0.00	C

HETATM	21 H10 NAP	1	0.867 -2.710	0.312 1.00 0.00	H

HETATM	22 C11 NAP	1	2.715 -1.681	0.080 1.00 0.00	C

HETATM	23 H11 NAP	1	3.328 -2.576	0.084 1.00 0.00	H

HETATM	24 C12 NAP	1	3.366 -0.425 -0.059 1.00 0.00	C

HETATM	25 C13 NAP	1	2.631	0.739 -0.070 1.00 0.00	C

HETATM	26 H13 NAP	1	3.106	1.707 -0.177 1.00 0.00	H

HETATM	27 O4 NAP	1	4.723 -0.501 -0.175 1.00 0.00	O

HETATM	28 C14 NAP	1	5.456	0.707 -0.318 1.00 0.00	C

HETATM	29 H14 NAP	1	6.502	0.414 -0.391 1.00 0.00	H

HETATM	30 H15 NAP	1	5.165	1.244 -1.227 1.00 0.00	H

HETATM	31 H16 NAP	1	5.321	1.359	0.550 1.00 0.00	H

CONECT	1	5	2	4	3

CONECT	2	1

CONECT	3	1

CONECT	4	1

CONECT	5	7	11	6	1

CONECT	6	5

CONECT	7	10	10	8	5

CONECT	8	9	7

CONECT	9	8

CONECT	10	7	7

CONECT	11	18	18	12	5

CONECT	12	14	14	11	13

CONECT	13	12

CONECT	14	15	20	12	12

CONECT	15	25	16	16	14

CONECT	16	17	15	15	18

CONECT	17	16

CONECT	18	16	19	11	11

CONECT	19	18

CONECT	20	22	22	14	21

CONECT	21	20

CONECT	22	24	23	20	20

CONECT	23	22

CONECT	24	27	25	25	22

CONECT	25	26	24	24	15

CONECT	26	25

CONECT	27	28	24

CONECT	28	30	29	27	31

CONECT	29	28

CONECT	30	28

CONECT	31	28

MASTER	0	0	0	0	0	0	0	0	31	0	31	0

END

Table S2. NPX^-^ PDB file

COMPND	Configuration number : 780L =	39.2084 39.2084 39.2084

AUTHOR	GENERATED BY OPEN BABEL 3.0.0

HETATM	1 C1 NAPH	1	-3.587 -0.134	1.803 1.00 0.00	C

HETATM	2 HC1 NAPH	1	-3.272 -1.177	1.865 1.00 0.00	H

HETATM	3 HC2 NAPH	1	-3.154	0.403	2.649 1.00 0.00	H

HETATM	4 HC3 NAPH	1	-4.675 -0.105	1.900 1.00 0.00	H

HETATM	5 C2 NAPH	1	-3.130	0.521	0.487 1.00 0.00	C

HETATM	6 H2 NAPH	1	-3.479	1.555	0.470 1.00 0.00	H

HETATM	7 C3 NAPH	1	-3.783 -0.112 -0.735 1.00 0.00	C

HETATM	8 OH NAPH	1	-3.730 -1.469 -0.713 1.00 0.00	O

HETATM	9 O3 NAPH	1	-4.293	0.493 -1.643 1.00 0.00	O

HETATM	10 C4 NAPH	1	-1.611	0.546	0.326 1.00 0.00	C

HETATM	11 C5 NAPH	1	-0.851 -0.607	0.331 1.00 0.00	C

HETATM	12 H51 NAPH	1	-1.332 -1.576	0.425 1.00 0.00	H

HETATM	13 C6 NAPH	1	0.558 -0.566	0.201 1.00 0.00	C

HETATM	14 C7 NAPH	1	1.215	0.693	0.059 1.00 0.00	C

HETATM	15 C8 NAPH	1	0.417	1.864	0.050 1.00 0.00	C

HETATM	16 H81 NAPH	1	0.899	2.832 -0.061 1.00 0.00	H

HETATM	17 C9 NAPH	1	-0.950	1.791	0.177 1.00 0.00	C

HETATM	18 H91 NAPH	1	-1.539	2.703	0.161 1.00 0.00	H

HETATM	19 C10 NAPH	1	1.355 -1.745	0.206 1.00 0.00	C

HETATM	20 H10 NAPH	1	0.867 -2.710	0.312 1.00 0.00	H

HETATM	21 C11 NAPH	1	2.715 -1.681	0.080 1.00 0.00	C

HETATM	22 H11 NAPH	1	3.328 -2.576	0.084 1.00 0.00	H

HETATM	23 C12 NAPH	1	3.366 -0.425 -0.059 1.00 0.00	C

HETATM	24 C13 NAPH	1	2.631	0.739 -0.070 1.00 0.00	C

HETATM	25 H13 NAPH	1	3.106	1.707 -0.177 1.00 0.00	H

HETATM	26 O4 NAPH	1	4.723 -0.501 -0.175 1.00 0.00	O

HETATM	27 C14 NAPH	1	5.456	0.707 -0.318 1.00 0.00	C

HETATM	28 H14 NAPH	1	6.502	0.414 -0.391 1.00 0.00	H

HETATM	29 H15 NAPH	1	5.165	1.244 -1.227 1.00 0.00	H

HETATM	30 H16 NAPH	1	5.321	1.359	0.550 1.00 0.00	H

CONECT	1	5	2	4	3

CONECT	2	1

CONECT	3	1

CONECT	4	1

CONECT	5	7	10	6	1

CONECT	6	5

CONECT	7	9	9	8	5

CONECT	8	7

CONECT	9	7	7

CONECT	10	17	17	11	5

CONECT	11	13	13	10	12

CONECT	12	11

CONECT	13	14	19	11	11

CONECT	14	24	15	15	13

CONECT	15	16	14	14	17

CONECT	16	15

CONECT	17	15	18	10	10

CONECT	18	17

CONECT	19	21	21	13	20

CONECT	20	19

CONECT	21	23	22	19	19

CONECT	22	21

CONECT	23	26	24	24	21

CONECT	24	25	23	23	14

CONECT	25	24

CONECT	26	27	23

CONECT	27	29	28	26	30

CONECT	28	27

CONECT	29	27

CONECT	30	27

MASTER	0	0	0	0	0	0	0	0	30	0	30	0

END

Table S3. NPX full force field (.itp file).

[ defaults ]

; nbfunc	comb-rule	gen-pairs	fudgeLJ fudgeQQ

1	3	yes	0.5	0.5

[ atomtypes ]

; name	bond_type at.num	mass	charge ptype sigma	epsilon

opls_135 CT	6 12.011000 -0.18000000 A	0.35	0.276144

opls_140 HC	1	1.008000 0.06000000 A	0.25	0.12552

opls_139 CT	6 12.011000 0.00000000 A	0.35	0.276144

opls_267 C	6 12.011000 0.52000000 A	0.375	0.43932

opls_268 OH	8 15.999400 -0.53000000 A	0.3	0.71128

opls_270 HO	1	1.008000 0.45000000 A	0	0

opls_269 O_3	8 15.999400 -0.44000000 A	0.296	0.87864

opls_145 CA	6 12.011000 -0.11500000 A	0.355	0.29288

opls_146 HA	1	1.008000 0.11500000 A	0.242	0.12552

opls_186 OS	8 15.999400 -0.40000000 A	0.29	0.58576

opls_181 CT	6 12.011000 0.11000000 A	0.35	0.276144

opls_185 HC	1	1.008000 0.03000000 A	0.25	0.12552

[ moleculetype ]

; Name	nrexcl

NAP	3

[ atoms ]

; nr	type resnr residue atom	cgnr	charge	mass typeB	chargeB	massB

; residue	1 NAP rtp NAP q 0.0

1	opls_135	1	NAP	C1	1 0.17900000 12.011000	; qtot 0.179000

2	opls_140	1	NAP	HC1	2 -0.02000000	1.008000	; qtot 0.159000

3	opls_140	1	NAP	HC2	3 -0.02000000	1.008000	; qtot 0.139000

4	opls_140	1	NAP	HC3	4 -0.02000000	1.008000	; qtot 0.119000

5	opls_139	1	NAP	C2	5 -0.24500000 12.011000	; qtot -0.126000

6	opls_140	1	NAP	H2	6 0.06300000	1.008000	; qtot -0.063000

7	opls_267	1	NAP	C3	7 0.97200000 12.011000	; qtot 0.909000

8	opls_268	1	NAP	OH	8 -0.57500000 15.999400	; qtot 0.334000

9	opls_270	1	NAP	H3	9 0.32400000	1.008000	; qtot 0.658000

10	opls_269	1	NAP	O3	10 -0.67300000 15.999400	; qtot -0.015000

11	opls_145	1	NAP	C4	11 -0.04900000 12.011000	; qtot -0.064000

12	opls_145	1	NAP	C5	12 -0.04900000 12.011000	; qtot -0.113000

13	opls_146	1	NAP	H51	13 0.08600000	1.008000	; qtot -0.027000

14	opls_145	1	NAP	C6	14 -0.04900000 12.011000	; qtot -0.076000

15	opls_145	1	NAP	C7	15 -0.04900000 12.011000	; qtot -0.125000

16	opls_145	1	NAP	C8	16 -0.04900000 12.011000	; qtot -0.174000

17	opls_146	1	NAP	H81	17 0.08600000	1.008000	; qtot -0.088000

18	opls_145	1	NAP	C9	18 -0.04900000 12.011000	; qtot -0.137000

19	opls_146	1	NAP	H91	19 0.08600000	1.008000	; qtot -0.051000

20	opls_145	1	NAP	C10	20 -0.04900000 12.011000	; qtot -0.100000

21	opls_146	1	NAP	H10	21 0.08600000	1.008000	; qtot -0.014000

22	opls_145	1	NAP	C11	22 -0.04900000 12.011000	; qtot -0.063000

23	opls_146	1	NAP	H11	23 0.08600000	1.008000	; qtot 0.023000

24	opls_145	1	NAP	C12	24 -0.04900000 12.011000	; qtot -0.026000

25	opls_145	1	NAP	C13	25 -0.04900000 12.011000	; qtot -0.075000

26	opls_146	1	NAP	H13	26 0.08600000	1.008000	; qtot 0.011000

27	opls_186	1	NAP	O4	27 -0.26600000 15.999400	; qtot -0.255000

28	opls_181	1	NAP	C14	28 0.26100000 12.011000	; qtot 0.006000

29	opls_185	1	NAP	H14	29 -0.00200000	1.008000	; qtot 0.004000

30	opls_185	1	NAP	H15	30 -0.00200000	1.008000	; qtot 0.002000

31	opls_185	1	NAP	H16	31 -0.00200000	1.008000	; qtot 0.000000

[ bonds ]

; ai	aj funct	c0	c1	c2	c3

1	2	1	0.10900 284512.000000

1	3	1	0.10900 284512.000000

1	4	1	0.10900 284512.000000

1	5	1	0.15290 224262.400000

5	6	1	0.10900 284512.000000

5	7	1	0.15220 265265.600000

5	11	1	0.15100 265265.600000

7	8	1	0.13640 376560.000000

7	10	1	0.12290 476976.000000

8	9	1	0.09450 462750.400000

11	12	1	0.14000 392459.200000

11	18	1	0.14000 392459.200000

12	13	1	0.10800 307105.600000

12	14	1	0.14000 392459.200000

14	15	1	0.14000 392459.200000

14	20	1	0.14000 392459.200000

15	16	1	0.14000 392459.200000

15	25	1	0.14000 392459.200000

16	17	1	0.10800 307105.600000

16	18	1	0.14000 392459.200000

18	19	1	0.10800 307105.600000

20	21	1	0.10800 307105.600000

20	22	1	0.14000 392459.200000

22	23	1	0.10800 307105.600000

22	24	1	0.14000 392459.200000

24	25	1	0.14000 392459.200000

24	27	1	0.13640 376560.000000

25	26	1	0.10800 307105.600000

27	28	1	0.14100 267776.000000

28	29	1	0.10900 284512.000000

28	30	1	0.10900 284512.000000

28	31	1	0.10900 284512.000000

[ pairs ]

; ai	aj funct	c0	c1	c2	c3

1	8	1

1	10	1

1	12	1

1	18	1

2	6	1

2	7	1

2	11	1

3	6	1

3	7	1

3	11	1

4	6	1

4	7	1

4	11	1

5	9	1

5	13	1

5	14	1

5	16	1

5	19	1

6	8	1

6	10	1

6	12	1

6	18	1

7	12	1

7	18	1

8	11	1

9	10	1

10	11	1

11	15	1

11	17	1

11	20	1

12	16	1

12	19	1

12	21	1

12	22	1

12	25	1

13	15	1

13	18	1

13	20	1

14	17	1

14	18	1

14	23	1

14	24	1

14	26	1

15	19	1

15	21	1

15	22	1

15	27	1

16	20	1

16	24	1

16	26	1

17	19	1

17	25	1

18	25	1

20	25	1

20	27	1

21	23	1

21	24	1

22	26	1

22	28	1

23	25	1

23	27	1

24	29	1

24	30	1

24	31	1

25	28	1

26	27	1

[ angles ]

; ai	aj	ak funct	c0	c1	c2	c3

2	1	3	1	107.8000000 276.144000

2	1	4	1	107.8000000 276.144000

2	1	5	1	110.7000000 313.800000

3	1	4	1	107.8000000 276.144000

3	1	5	1	110.7000000 313.800000

4	1	5	1	110.7000000 313.800000

1	5	6	1	110.7000000 313.800000

1	5	7	1	111.1000000 527.184000

1	5	11	1	114.0000000 527.184000

6	5	7	1	109.5000000 292.880000

6	5	11	1	109.5000000 292.880000

7	5	11	1	112.0000000 527.184000

5	7	8	1	108.0000000 585.760000

5	7	10	1	120.4000000 669.440000

8	7	10	1	121.0000000 669.440000

7	8	9	1	113.0000000 292.880000

5	11	12	1	120.0000000 585.760000

5	11	18	1	120.0000000 585.760000

12	11	18	1	120.0000000 527.184000

11	12	13	1	120.0000000 292.880000

11	12	14	1	120.0000000 527.184000

13	12	14	1	120.0000000 292.880000

12	14	15	1	120.0000000 527.184000

12	14	20	1	120.0000000 527.184000

15	14	20	1	120.0000000 527.184000

14	15	16	1	120.0000000 527.184000

14	15	25	1	120.0000000 527.184000

16	15	25	1	120.0000000 527.184000

15	16	17	1	120.0000000 292.880000

15	16	18	1	120.0000000 527.184000

17	16	18	1	120.0000000 292.880000

11	18	16	1	120.0000000 527.184000

11	18	19	1	120.0000000 292.880000

16	18	19	1	120.0000000 292.880000

14	20	21	1	120.0000000 292.880000

14	20	22	1	120.0000000 527.184000

21	20	22	1	120.0000000 292.880000

20	22	23	1	120.0000000 292.880000

20	22	24	1	120.0000000 527.184000

23	22	24	1	120.0000000 292.880000

22	24	25	1	120.0000000 527.184000

22	24	27	1	120.0000000 585.760000

25	24	27	1	120.0000000 585.760000

15	25	24	1	120.0000000 527.184000

15	25	26	1	120.0000000 292.880000

24	25	26	1	120.0000000 292.880000

24	27	28	1	111.0000000 627.600000

27	28	29	1	109.5000000 292.880000

27	28	30	1	109.5000000 292.880000

27	28	31	1	109.5000000 292.880000

29	28	30	1	107.8000000 276.144000

29	28	31	1	107.8000000 276.144000

30	28	31	1	107.8000000 276.144000

[ dihedrals ]

; ai	aj	ak	al funct	c0	c1	c2	c3	c4	c5

5	8	7	10	1 180.0000000 43.9320000 2

5	11	12	18	1 180.0000000 4.6024000 2

11	14	12	13	1 180.0000000 4.6024000 2

12	15	14	20	1 180.0000000 4.6024000 2

14	22	20	21	1 180.0000000 4.6024000 2

14	16	15	25	1 180.0000000 4.6024000 2

15	18	16	17	1 180.0000000 4.6024000 2

16	11	18	19	1 180.0000000 4.6024000 2

20	24	22	23	1 180.0000000 4.6024000 2

22	25	24	27	1 180.0000000 4.6024000 2

24	15	25	26	1 180.0000000 4.6024000 2

[ dihedrals ]

; ai	aj	ak	al funct	c0	c1	c2	c3	c4	c5

2	1	5	6	3	0.6276000	1.8828000	0.0000000	-2.5104000	0.0000000	0.0000000

2	1	5	7	3	-0.2092000	-0.6276000	0.0000000	0.8368000	0.0000000	0.0000000

2	1	5	11	3	0.9665000	2.8995100	0.0000000	-3.8660100	0.0000000	0.0000000

3	1	5	6	3	0.6276000	1.8828000	0.0000000	-2.5104000	0.0000000	0.0000000

3	1	5	7	3	-0.2092000	-0.6276000	0.0000000	0.8368000	0.0000000	0.0000000

3	1	5	11	3	0.9665000	2.8995100	0.0000000	-3.8660100	0.0000000	0.0000000

4	1	5	6	3	0.6276000	1.8828000	0.0000000	-2.5104000	0.0000000	0.0000000

4	1	5	7	3	-0.2092000	-0.6276000	0.0000000	0.8368000	0.0000000	0.0000000

4	1	5	11	3	0.9665000	2.8995100	0.0000000	-3.8660100	0.0000000	0.0000000

11	5	7	8	3	3.1298000	3.4583000	-1.2273000	0.8688000	0.2895000	0.0000000

11	5	7	10	3	3.1298000	3.4583000	-1.2273000	0.8688000	0.2895000	0.0000000

1	5	7	8	3	5.3178600	0.7322000	-2.2844600	-3.7656000	0.0000000	0.0000000

1	5	7	10	3	2.2844600	0.0000000	-2.2844600	0.0000000	0.0000000	0.0000000

6	5	7	8	3	0.0000000	0.0000000	0.0000000	0.0000000	0.0000000	0.0000000

6	5	7	10	3	0.0000000	0.0000000	0.0000000	0.0000000	0.0000000	0.0000000

1	5	11	12	3	0.0000000	0.0000000	0.0000000	0.0000000	0.0000000	0.0000000

1	5	11	18	3	0.0000000	0.0000000	0.0000000	0.0000000	0.0000000	0.0000000

6	5	11	12	3	0.0000000	0.0000000	0.0000000	0.0000000	0.0000000	0.0000000

6	5	11	18	3	0.0000000	0.0000000	0.0000000	0.0000000	0.0000000	0.0000000

7	5	11	12	3	0.0000000	0.0000000	0.0000000	0.0000000	0.0000000	0.0000000

7	5	11	18	3	0.0000000	0.0000000	0.0000000	0.0000000	0.0000000	0.0000000

5	7	8	9	3	26.1500000	-3.1380000	-23.0120000	0.0000000	0.0000000	0.0000000

10	7	8	9	3	23.0120000	0.0000000	-23.0120000	0.0000000	0.0000000	0.0000000

5	11	12	13	3	30.3340000	0.0000000	-30.3340000	0.0000000	0.0000000	0.0000000

5	11	12	14	3	30.3340000	0.0000000	-30.3340000	0.0000000	0.0000000	0.0000000

18	11	12	13	3	30.3340000	0.0000000	-30.3340000	0.0000000	0.0000000	0.0000000

18	11	12	14	3	30.3340000	0.0000000	-30.3340000	0.0000000	0.0000000	0.0000000

5	11	18	16	3	30.3340000	0.0000000	-30.3340000	0.0000000	0.0000000	0.0000000

5	11	18	19	3	30.3340000	0.0000000	-30.3340000	0.0000000	0.0000000	0.0000000

12	11	18	16	3	30.3340000	0.0000000	-30.3340000	0.0000000	0.0000000	0.0000000

12	11	18	19	3	30.3340000	0.0000000	-30.3340000	0.0000000	0.0000000	0.0000000

11	12	14	15	3	30.3340000	0.0000000	-30.3340000	0.0000000	0.0000000	0.0000000

11	12	14	20	3	30.3340000	0.0000000	-30.3340000	0.0000000	0.0000000	0.0000000

13	12	14	15	3	30.3340000	0.0000000	-30.3340000	0.0000000	0.0000000	0.0000000

13	12	14	20	3	30.3340000	0.0000000	-30.3340000	0.0000000	0.0000000	0.0000000

12	14	15	16	3	30.3340000	0.0000000	-30.3340000	0.0000000	0.0000000	0.0000000

12	14	15	25	3	30.3340000	0.0000000	-30.3340000	0.0000000	0.0000000	0.0000000

20	14	15	16	3	30.3340000	0.0000000	-30.3340000	0.0000000	0.0000000	0.0000000

20	14	15	25	3	30.3340000	0.0000000	-30.3340000	0.0000000	0.0000000	0.0000000

12	14	20	21	3	30.3340000	0.0000000	-30.3340000	0.0000000	0.0000000	0.0000000

12	14	20	22	3	30.3340000	0.0000000	-30.3340000	0.0000000	0.0000000	0.0000000

15	14	20	21	3	30.3340000	0.0000000	-30.3340000	0.0000000	0.0000000	0.0000000

15	14	20	22	3	30.3340000	0.0000000	-30.3340000	0.0000000	0.0000000	0.0000000

14	15	16	17	3	30.3340000	0.0000000	-30.3340000	0.0000000	0.0000000	0.0000000

14	15	16	18	3	30.3340000	0.0000000	-30.3340000	0.0000000	0.0000000	0.0000000

25	15	16	17	3	30.3340000	0.0000000	-30.3340000	0.0000000	0.0000000	0.0000000

25	15	16	18	3	30.3340000	0.0000000	-30.3340000	0.0000000	0.0000000	0.0000000

14	15	25	24	3	30.3340000	0.0000000	-30.3340000	0.0000000	0.0000000	0.0000000

14	15	25	26	3	30.3340000	0.0000000	-30.3340000	0.0000000	0.0000000	0.0000000

16	15	25	24	3	30.3340000	0.0000000	-30.3340000	0.0000000	0.0000000	0.0000000

16	15	25	26	3	30.3340000	0.0000000	-30.3340000	0.0000000	0.0000000	0.0000000

15	16	18	11	3	30.3340000	0.0000000	-30.3340000	0.0000000	0.0000000	0.0000000

15	16	18	19	3	30.3340000	0.0000000	-30.3340000	0.0000000	0.0000000	0.0000000

17	16	18	11	3	30.3340000	0.0000000	-30.3340000	0.0000000	0.0000000	0.0000000

17	16	18	19	3	30.3340000	0.0000000	-30.3340000	0.0000000	0.0000000	0.0000000

14	20	22	23	3	30.3340000	0.0000000	-30.3340000	0.0000000	0.0000000	0.0000000

14	20	22	24	3	30.3340000	0.0000000	-30.3340000	0.0000000	0.0000000	0.0000000

21	20	22	23	3	30.3340000	0.0000000	-30.3340000	0.0000000	0.0000000	0.0000000

21	20	22	24	3	30.3340000	0.0000000	-30.3340000	0.0000000	0.0000000	0.0000000

20	22	24	25	3	30.3340000	0.0000000	-30.3340000	0.0000000	0.0000000	0.0000000

20	22	24	27	3	30.3340000	0.0000000	-30.3340000	0.0000000	0.0000000	0.0000000

23	22	24	25	3	30.3340000	0.0000000	-30.3340000	0.0000000	0.0000000	0.0000000

23	22	24	27	3	30.3340000	0.0000000	-30.3340000	0.0000000	0.0000000	0.0000000

22	24	25	15	3	30.3340000	0.0000000	-30.3340000	0.0000000	0.0000000	0.0000000

22	24	25	26	3	30.3340000	0.0000000	-30.3340000	0.0000000	0.0000000	0.0000000

27	24	25	15	3	30.3340000	0.0000000	-30.3340000	0.0000000	0.0000000	0.0000000

27	24	25	26	3	30.3340000	0.0000000	-30.3340000	0.0000000	0.0000000	0.0000000

22	24	27	28	3	12.5520000	0.0000000	-12.5520000	0.0000000	0.0000000	0.0000000

25	24	27	28	3	12.5520000	0.0000000	-12.5520000	0.0000000	0.0000000	0.0000000

24	27	28	29	3	1.5899200	4.7697600	0.0000000	-6.3596800	0.0000000	0.0000000

24	27	28	30	3	1.5899200	4.7697600	0.0000000	-6.3596800	0.0000000	0.0000000

24	27	28	31	3	1.5899200	4.7697600	0.0000000	-6.3596800	0.0000000	0.0000000

[ system ]

; Name

Generic title

[ molecules ]

; Compound	#mols

NAP	1

Table S4. NPX^-^ full force field (.itp file).

[ defaults ]

; nbfunc	comb-rule	gen-pairs	fudgeLJ fudgeQQ

1	3	yes	0.5	0.5

[ atomtypes ]

; name	bond_type at.num	mass	charge ptype sigma	epsilon

opls_135 CT	6 12.011000 -0.18000000 A	0.35	0.276144

opls_140 HC	1	1.008000 0.06000000 A	0.25	0.12552

opls_139 CT	6 12.011000 0.00000000 A	0.35	0.276144

opls_267 C	6 12.011000 0.52000000 A	0.375	0.43932

opls_268 OH	8 15.999400 -0.53000000 A	0.3	0.71128

opls_269 O_3	8 15.999400 -0.44000000 A	0.296	0.87864

opls_145 CA	6 12.011000 -0.11500000 A	0.355	0.29288

opls_146 HA	1	1.008000 0.11500000 A	0.242	0.12552

opls_186 OS	8 15.999400 -0.40000000 A	0.29	0.58576

opls_181 CT	6 12.011000 0.11000000 A	0.35	0.276144

opls_185 HC	1	1.008000 0.03000000 A	0.25	0.12552

[ moleculetype ]

; Name	nrexcl

NAPH	3

[ atoms ]

; nr	type resnr residue atom	cgnr	charge	mass typeB	chargeB	massB

; residue	1 NAPH rtp NAPH q -1.0

1	opls_135	1	NAPH	C1	1 -0.13600000 12.011000	; qtot -0.136000

2	opls_140	1	NAPH	HC1	2 -0.00800000	1.008000	; qtot -0.144000

3	opls_140	1	NAPH	HC2	3 -0.00800000	1.008000	; qtot -0.152000

4	opls_140	1	NAPH	HC3	4 -0.00800000	1.008000	; qtot -0.160000

5	opls_139	1	NAPH	C2	5 0.55700000 12.011000	; qtot 0.397000

6	opls_140	1	NAPH	H2	6 -0.19300000	1.008000	; qtot 0.204000

7	opls_267	1	NAPH	C3	7 0.55900000 12.011000	; qtot 0.763000

8	opls_268	1	NAPH	OH	8 -0.77900000 15.999400	; qtot -0.016000

9	opls_269	1	NAPH	O3	9 -0.77900000 15.999400	; qtot -0.795000

10	opls_145	1	NAPH	C4	10 -0.06500000 12.011000	; qtot -0.860000

11	opls_145	1	NAPH	C5	11 -0.06500000 12.011000	; qtot -0.925000

12	opls_146	1	NAPH	H51	12 0.08200000	1.008000	; qtot -0.843000

13	opls_145	1	NAPH	C6	13 -0.06500000 12.011000	; qtot -0.908000

14	opls_145	1	NAPH	C7	14 -0.06500000 12.011000	; qtot -0.973000

15	opls_145	1	NAPH	C8	15 -0.06500000 12.011000	; qtot -1.038000

16	opls_146	1	NAPH	H81	16 0.08200000	1.008000	; qtot -0.956000

17	opls_145	1	NAPH	C9	17 -0.06500000 12.011000	; qtot -1.021000

18	opls_146	1	NAPH	H91	18 0.08200000	1.008000	; qtot -0.939000

19	opls_145	1	NAPH	C10	19 -0.06500000 12.011000	; qtot -1.004000

20	opls_146	1	NAPH	H10	20 0.08200000	1.008000	; qtot -0.922000

21	opls_145	1	NAPH	C11	21 -0.06500000 12.011000	; qtot -0.987000

22	opls_146	1	NAPH	H11	22 0.08200000	1.008000	; qtot -0.905000

23	opls_145	1	NAPH	C12	23 -0.06500000 12.011000	; qtot -0.970000

24	opls_145	1	NAPH	C13	24 -0.06500000 12.011000	; qtot -1.035000

25	opls_146	1	NAPH	H13	25 0.08200000	1.008000	; qtot -0.953000

26	opls_186	1	NAPH	O4	26 -0.26600000 15.999400	; qtot -1.219000

27	opls_181	1	NAPH	C14	27 0.23700000 12.011000	; qtot -0.982000

28	opls_185	1	NAPH	H14	28 -0.00600000	1.008000	; qtot -0.988000

29	opls_185	1	NAPH	H15	29 -0.00600000	1.008000	; qtot -0.994000

30	opls_185	1	NAPH	H16	30 -0.00600000	1.008000	; qtot -1.000000

[ bonds ]

; ai	aj funct	c0	c1	c2	c3

1	2	1	0.10900 284512.000000

1	3	1	0.10900 284512.000000

1	4	1	0.10900 284512.000000

1	5	1	0.15290 224262.400000

5	6	1	0.10900 284512.000000

5	7	1	0.15220 265265.600000

5	10	1	0.15100 265265.600000

7	8	1	0.13640 376560.000000

7	9	1	0.12290 476976.000000

10	11	1	0.14000 392459.200000

10	17	1	0.14000 392459.200000

11	12	1	0.10800 307105.600000

11	13	1	0.14000 392459.200000

13	14	1	0.14000 392459.200000

13	19	1	0.14000 392459.200000

14	15	1	0.14000 392459.200000

14	24	1	0.14000 392459.200000

15	16	1	0.10800 307105.600000

15	17	1	0.14000 392459.200000

17	18	1	0.10800 307105.600000

19	20	1	0.10800 307105.600000

19	21	1	0.14000 392459.200000

21	22	1	0.10800 307105.600000

21	23	1	0.14000 392459.200000

23	24	1	0.14000 392459.200000

23	26	1	0.13640 376560.000000

24	25	1	0.10800 307105.600000

26	27	1	0.14100 267776.000000

27	28	1	0.10900 284512.000000

27	29	1	0.10900 284512.000000

27	30	1	0.10900 284512.000000

[ pairs ]

; ai	aj funct	c0	c1	c2	c3

1	8	1

1	9	1

1	11	1

1	17	1

2	6	1

2	7	1

2	10	1

3	6	1

3	7	1

3	10	1

4	6	1

4	7	1

4	10	1

5	12	1

5	13	1

5	15	1

5	18	1

6	8	1

6	9	1

6	11	1

6	17	1

7	11	1

7	17	1

8	10	1

9	10	1

10	14	1

10	16	1

10	19	1

11	15	1

11	18	1

11	20	1

11	21	1

11	24	1

12	14	1

12	17	1

12	19	1

13	16	1

13	17	1

13	22	1

13	23	1

13	25	1

14	18	1

14	20	1

14	21	1

14	26	1

15	19	1

15	23	1

15	25	1

16	18	1

16	24	1

17	24	1

19	24	1

19	26	1

20	22	1

20	23	1

21	25	1

21	27	1

22	24	1

22	26	1

23	28	1

23	29	1

23	30	1

24	27	1

25	26	1

[ angles ]

; ai	aj	ak funct	c0	c1	c2	c3

2	1	3	1	107.8000000 276.144000

2	1	4	1	107.8000000 276.144000

2	1	5	1	110.7000000 313.800000

3	1	4	1	107.8000000 276.144000

3	1	5	1	110.7000000 313.800000

4	1	5	1	110.7000000 313.800000

1	5	6	1	110.7000000 313.800000

1	5	7	1	111.1000000 527.184000

1	5	10	1	114.0000000 527.184000

6	5	7	1	109.5000000 292.880000

6	5	10	1	109.5000000 292.880000

7	5	10	1	112.0000000 527.184000

5	7	8	1	108.0000000 585.760000

5	7	9	1	120.4000000 669.440000

8	7	9	1	121.0000000 669.440000

5	10	11	1	120.0000000 585.760000

5	10	17	1	120.0000000 585.760000

11	10	17	1	120.0000000 527.184000

10	11	12	1	120.0000000 292.880000

10	11	13	1	120.0000000 527.184000

12	11	13	1	120.0000000 292.880000

11	13	14	1	120.0000000 527.184000

11	13	19	1	120.0000000 527.184000

14	13	19	1	120.0000000 527.184000

13	14	15	1	120.0000000 527.184000

13	14	24	1	120.0000000 527.184000

15	14	24	1	120.0000000 527.184000

14	15	16	1	120.0000000 292.880000

14	15	17	1	120.0000000 527.184000

16	15	17	1	120.0000000 292.880000

10	17	15	1	120.0000000 527.184000

10	17	18	1	120.0000000 292.880000

15	17	18	1	120.0000000 292.880000

13	19	20	1	120.0000000 292.880000

13	19	21	1	120.0000000 527.184000

20	19	21	1	120.0000000 292.880000

19	21	22	1	120.0000000 292.880000

19	21	23	1	120.0000000 527.184000

22	21	23	1	120.0000000 292.880000

21	23	24	1	120.0000000 527.184000

21	23	26	1	120.0000000 585.760000

24	23	26	1	120.0000000 585.760000

14	24	23	1	120.0000000 527.184000

14	24	25	1	120.0000000 292.880000

23	24	25	1	120.0000000 292.880000

23	26	27	1	111.0000000 627.600000

26	27	28	1	109.5000000 292.880000

26	27	29	1	109.5000000 292.880000

26	27	30	1	109.5000000 292.880000

28	27	29	1	107.8000000 276.144000

28	27	30	1	107.8000000 276.144000

29	27	30	1	107.8000000 276.144000

[ dihedrals ]

; ai	aj	ak	al funct	c0	c1	c2	c3	c4	c5

5	8	7	9	1 180.0000000 43.9320000 2

5	10	11	17	1 180.0000000 4.6024000 2

10	13	11	12	1 180.0000000 4.6024000 2

11	14	13	19	1 180.0000000 4.6024000 2

13	21	19	20	1 180.0000000 4.6024000 2

13	15	14	24	1 180.0000000 4.6024000 2

14	17	15	16	1 180.0000000 4.6024000 2

15	10	17	18	1 180.0000000 4.6024000 2

19	23	21	22	1 180.0000000 4.6024000 2

21	24	23	26	1 180.0000000 4.6024000 2

23	14	24	25	1 180.0000000 4.6024000 2

[ dihedrals ]

; ai	aj	ak	al funct	c0	c1	c2	c3	c4	c5

2	1	5	6	3	0.6276000	1.8828000	0.0000000	-2.5104000	0.0000000	0.0000000

2	1	5	7	3	-0.2092000	-0.6276000	0.0000000	0.8368000	0.0000000	0.0000000

2	1	5	10	3	0.9665000	2.8995100	0.0000000	-3.8660100	0.0000000	0.0000000

3	1	5	6	3	0.6276000	1.8828000	0.0000000	-2.5104000	0.0000000	0.0000000

3	1	5	7	3	-0.2092000	-0.6276000	0.0000000	0.8368000	0.0000000	0.0000000

3	1	5	10	3	0.9665000	2.8995100	0.0000000	-3.8660100	0.0000000	0.0000000

4	1	5	6	3	0.6276000	1.8828000	0.0000000	-2.5104000	0.0000000	0.0000000

4	1	5	7	3	-0.2092000	-0.6276000	0.0000000	0.8368000	0.0000000	0.0000000

4	1	5	10	3	0.9665000	2.8995100	0.0000000	-3.8660100	0.0000000	0.0000000

10	5	7	8	3	3.1298000	3.4583000	-1.2273000	0.8688000	0.2895000	0.0000000

10	5	7	9	3	3.1298000	3.4583000	-1.2273000	0.8688000	0.2895000	0.0000000

1	5	7	8	3	5.3178600	0.7322000	-2.2844600	-3.7656000	0.0000000	0.0000000

1	5	7	9	3	2.2844600	0.0000000	-2.2844600	0.0000000	0.0000000	0.0000000

6	5	7	8	3	0.0000000	0.0000000	0.0000000	0.0000000	0.0000000	0.0000000

6	5	7	9	3	0.0000000	0.0000000	0.0000000	0.0000000	0.0000000	0.0000000

1	5	10	11	3	0.0000000	0.0000000	0.0000000	0.0000000	0.0000000	0.0000000

1	5	10	17	3	0.0000000	0.0000000	0.0000000	0.0000000	0.0000000	0.0000000

6	5	10	11	3	0.0000000	0.0000000	0.0000000	0.0000000	0.0000000	0.0000000

6	5	10	17	3	0.0000000	0.0000000	0.0000000	0.0000000	0.0000000	0.0000000

7	5	10	11	3	0.0000000	0.0000000	0.0000000	0.0000000	0.0000000	0.0000000

7	5	10	17	3	0.0000000	0.0000000	0.0000000	0.0000000	0.0000000	0.0000000

5	10	11	12	3	30.3340000	0.0000000	-30.3340000	0.0000000	0.0000000	0.0000000

5	10	11	13	3	30.3340000	0.0000000	-30.3340000	0.0000000	0.0000000	0.0000000

17	10	11	12	3	30.3340000	0.0000000	-30.3340000	0.0000000	0.0000000	0.0000000

17	10	11	13	3	30.3340000	0.0000000	-30.3340000	0.0000000	0.0000000	0.0000000

5	10	17	15	3	30.3340000	0.0000000	-30.3340000	0.0000000	0.0000000	0.0000000

5	10	17	18	3	30.3340000	0.0000000	-30.3340000	0.0000000	0.0000000	0.0000000

11	10	17	15	3	30.3340000	0.0000000	-30.3340000	0.0000000	0.0000000	0.0000000

11	10	17	18	3	30.3340000	0.0000000	-30.3340000	0.0000000	0.0000000	0.0000000

10	11	13	14	3	30.3340000	0.0000000	-30.3340000	0.0000000	0.0000000	0.0000000

10	11	13	19	3	30.3340000	0.0000000	-30.3340000	0.0000000	0.0000000	0.0000000

12	11	13	14	3	30.3340000	0.0000000	-30.3340000	0.0000000	0.0000000	0.0000000

12	11	13	19	3	30.3340000	0.0000000	-30.3340000	0.0000000	0.0000000	0.0000000

11	13	14	15	3	30.3340000	0.0000000	-30.3340000	0.0000000	0.0000000	0.0000000

11	13	14	24	3	30.3340000	0.0000000	-30.3340000	0.0000000	0.0000000	0.0000000

19	13	14	15	3	30.3340000	0.0000000	-30.3340000	0.0000000	0.0000000	0.0000000

19	13	14	24	3	30.3340000	0.0000000	-30.3340000	0.0000000	0.0000000	0.0000000

11	13	19	20	3	30.3340000	0.0000000	-30.3340000	0.0000000	0.0000000	0.0000000

11	13	19	21	3	30.3340000	0.0000000	-30.3340000	0.0000000	0.0000000	0.0000000

14	13	19	20	3	30.3340000	0.0000000	-30.3340000	0.0000000	0.0000000	0.0000000

14	13	19	21	3	30.3340000	0.0000000	-30.3340000	0.0000000	0.0000000	0.0000000

13	14	15	16	3	30.3340000	0.0000000	-30.3340000	0.0000000	0.0000000	0.0000000

13	14	15	17	3	30.3340000	0.0000000	-30.3340000	0.0000000	0.0000000	0.0000000

24	14	15	16	3	30.3340000	0.0000000	-30.3340000	0.0000000	0.0000000	0.0000000

24	14	15	17	3	30.3340000	0.0000000	-30.3340000	0.0000000	0.0000000	0.0000000

13	14	24	23	3	30.3340000	0.0000000	-30.3340000	0.0000000	0.0000000	0.0000000

13	14	24	25	3	30.3340000	0.0000000	-30.3340000	0.0000000	0.0000000	0.0000000

15	14	24	23	3	30.3340000	0.0000000	-30.3340000	0.0000000	0.0000000	0.0000000

15	14	24	25	3	30.3340000	0.0000000	-30.3340000	0.0000000	0.0000000	0.0000000

14	15	17	10	3	30.3340000	0.0000000	-30.3340000	0.0000000	0.0000000	0.0000000

14	15	17	18	3	30.3340000	0.0000000	-30.3340000	0.0000000	0.0000000	0.0000000

16	15	17	10	3	30.3340000	0.0000000	-30.3340000	0.0000000	0.0000000	0.0000000

16	15	17	18	3	30.3340000	0.0000000	-30.3340000	0.0000000	0.0000000	0.0000000

13	19	21	22	3	30.3340000	0.0000000	-30.3340000	0.0000000	0.0000000	0.0000000

13	19	21	23	3	30.3340000	0.0000000	-30.3340000	0.0000000	0.0000000	0.0000000

20	19	21	22	3	30.3340000	0.0000000	-30.3340000	0.0000000	0.0000000	0.0000000

20	19	21	23	3	30.3340000	0.0000000	-30.3340000	0.0000000	0.0000000	0.0000000

19	21	23	24	3	30.3340000	0.0000000	-30.3340000	0.0000000	0.0000000	0.0000000

19	21	23	26	3	30.3340000	0.0000000	-30.3340000	0.0000000	0.0000000	0.0000000

22	21	23	24	3	30.3340000	0.0000000	-30.3340000	0.0000000	0.0000000	0.0000000

22	21	23	26	3	30.3340000	0.0000000	-30.3340000	0.0000000	0.0000000	0.0000000

21	23	24	14	3	30.3340000	0.0000000	-30.3340000	0.0000000	0.0000000	0.0000000

21	23	24	25	3	30.3340000	0.0000000	-30.3340000	0.0000000	0.0000000	0.0000000

26	23	24	14	3	30.3340000	0.0000000	-30.3340000	0.0000000	0.0000000	0.0000000

26	23	24	25	3	30.3340000	0.0000000	-30.3340000	0.0000000	0.0000000	0.0000000

21	23	26	27	3	12.5520000	0.0000000	-12.5520000	0.0000000	0.0000000	0.0000000

24	23	26	27	3	12.5520000	0.0000000	-12.5520000	0.0000000	0.0000000	0.0000000

23	26	27	28	3	1.5899200	4.7697600	0.0000000	-6.3596800	0.0000000	0.0000000

23	26	27	29	3	1.5899200	4.7697600	0.0000000	-6.3596800	0.0000000	0.0000000

23	26	27	30	3	1.5899200	4.7697600	0.0000000	-6.3596800	0.0000000	0.0000000

[ system ]

; Name

Generic title

[ molecules ]

; Compound	#mols

NAPH	1

|  |  |
| --- | --- |
| (a) | (d) |
|  |  |
| (b) | (e) |
|  |  |
| (c) | (f) |

Figure S1 – NXP (a) and NPX^-^ (d) structure with the identification of dihedral atoms. Saved structures conformation of molecular dynamics in NPX in ACN (b), ETH (c), and NPX^-^ in ACN(e) and ETH (f).

|  |  |
| --- | --- |
| (a) | (b) |
|  |  |
| (c) | (d) |

Figure S2 – Evaluation of solvent influence in NPX ECD spectra. Changing the Pople, Dunning and Karlsruhe bases set in front of DFT functional: B3LYP (a), CAM-B3LYP (b), M06-2X (c) and *ω*B97XD (d). All analysis were performed in TD(NStates=15).

|  |  |
| --- | --- |
| (a) | (e) |
|  |  |
| (b) | (f) |
|  |  |
| (c) | (g) |
|  |  |
| (d) | (h) |

Figure S3 – All individual ECD spectra to each cluster of NPX GAS (a), WAT (b), ACN (c), ETH (d) and to the NPX^-^ GAS (e), WAT(f), ACN (g), ETH (h). The CAM-B3LYP/aug-cc-pVDZ/ TD(NStates=15) level was used.

|  |  |
| --- | --- |
| (a) | (b) |
|  |  |
| (c) | (d) |

Figure S4 – Theoretical ECD spectra of NPX and NPX⁻ in the gas phase (a). NPX in ACN (b), WAT (c), and ETH (d) – computed the cluster isolated from solvent (gas phase), the first and second solvation shells. The CAM-B3LYP/aug-cc-pVDZ/ TD(NStates=15) level was used.

|  |  |
| --- | --- |
| (a) | (b) |
|  | |
| (C) | |

Figure S5 – Comparison of the ECD spectra with NStates=15 and NStates=30 for NPX^-^ in ACN (a), WAT (b), and ETH (c). The CAM-B3LYP/aug-cc-pVDZ/ level was used.

|  |  |
| --- | --- |
| (a) | (b) |
|  |  |
| (c) | (d) |

Figure S6 – Resulting dipole moment to the NPX/ACN (a), NPX^-^/ACN (b), NPX/ETH (c) and NPX^-^/ETH system.

|  |  |
| --- | --- |
| (a) | (b) |
|  |  |
| (c) | (d) |
|  |  |
| (e) | (f) |

Figure S7 – Density Transition Difference Iso-surface of NPX via EDD analysis (Iso-values=0.001). Cluster 1: ACN (a) – GAS (b). Cluster 2: ACN (c) – GAS (d). Cluster 3: ACN (e) – GAS (f).

|  |  |
| --- | --- |
| (a) | (b) |
|  |  |
| (c) | (e) |
|  |  |
| (f) | (g) |

Figure S8 – Density Transition Difference Iso-surface of NPX via EDD analysis (Iso-values=0.001). Cluster 1: ETH (a) – GAS (b). Cluster 2: ETH (c) – GAS (d). Cluster 3: ETH (e) – GAS (f).

|  |  |
| --- | --- |
| (a) | (b) |
|  |  |
| (c) | (d) |
|  |  |
| (e) | (f) |

Figure S9 – Density Transition Difference Iso-surface of NPX via EDD analysis (Iso-values=0.001). Cluster 1: WAT (a) – GAS (b). Cluster 2: WAT (c) – GAS (d). Cluster 3: WAT (e) – GAS (f).

|  |  |
| --- | --- |
| (a) | (b) |
|  |  |
| (c) | (d) |
|  |  |
| (e) | (f) |

Figure S10 – Density Transition Difference Iso-surface of NPX^-^ via EDD analysis (Iso-values=0.001). Cluster 1: ACN (a) – GAS (b). Cluster 2: ACN (c) – GAS (d). Cluster 3: ACN (e) – GAS (f).

|  |  |
| --- | --- |
| (a) | (b) |
|  |  |
| (c) | (d) |
|  |  |
| (e) | (f) |

Figure S11 – Density Transition Difference Iso-surface of NPX^-^ via EDD analysis (Iso-values=0.001). Cluster 1: ETH (a) – GAS (b). Cluster 2: ETH (c) – GAS (d). Cluster 3: ETH (e) – GAS (f).

|  |  |
| --- | --- |
| (a) | (b) |
|  |  |
| (c) | (d) |
|  |  |
| (e) | (f) |

Figure S12 – Density Transition Difference Iso-surface of NPX^-^ via EDD analysis (Iso-values=0.001). Cluster 1: WAT (a) – GAS (b). Cluster 2: WAT (c) – GAS (d). Cluster 3: WAT (e) – GAS (f)

Table S5– Isotropic polarizability ($\alpha$) and total first hyperpolarizability ($\beta_{tot}$) of neutral (NPX) and anionic (NPX^-^) naproxen clusters in gas and solvent phases.

| System | | | Polarizability  Å^3^ | Hyperpolarizability  10^-33^ esu |
| --- | --- | --- | --- | --- |
| NPX | Cluster 01 | Gas | 27.6313 | 5419.9945 |
|  |  | ACN (2SS) | 70.1307 | 4197.8275 |
|  | Cluster 02 | Gas | 27.9022 | 6001.9715 |
|  |  | ACN (2SS) | 69.8302 | 3039.0402 |
|  | Cluster 03 | Gas | 27.7171 | 5110.9871 |
|  |  | ACN (2SS) | 69.3042 | 2098.4553 |
| NPX^-^ | Cluster 01 | Gas | 29.6141 | 9033.9735 |
|  |  | ACN (2SS) | 71.6627 | 3760.7478 |
|  | Cluster 02 | Gas | 29.5560 | 8737.8222 |
|  |  | ACN (2SS) | 71.7265 | 5097.4988 |
|  | Cluster 03 | Gas | 29.6506 | 5938.4945 |
|  |  | ACN (2SS) | 72.7598 | 1748.6719 |
| NPX | Cluster 01 | Gas | 27.9123 | 5793.1477 |
|  |  | ETH (2SS) | 75.8563 | 6378.0421 |
|  | Cluster 02 | Gas | 28.0569 | 5659.8014 |
|  |  | ETH (2SS) | 75.1141 | 3059.1328 |
|  | Cluster 03 | Gas | 27.7730 | 4693.6711 |
|  |  | ETH (2SS) | 74.9947 | 3270.3159 |
| NPX^-^ | Cluster 01 | Gas | 29.7767 | 11302.2567 |
|  |  | ETH (2SS) | 77.0904 | 5502.3092 |
|  | Cluster 02 | Gas | 29.6032 | 10104.0012 |
|  |  | ETH (2SS) | 77.1139 | 3366.9159 |
|  | Cluster 03 | Gas | 29.8491 | 9307.9125 |
|  |  | ETH (2SS) | 76.7937 | 6606.5142 |

|  |  |
| --- | --- |
| (a) | (b) |
|  |  |
| (c) | (d) |
|  |  |
| (e) | (f) |
|  |  |
| (g) | (h) |
|  |  |
| (i) | (j) |
|  |  |
| (k) | (l) |

Figure S13 – NPX NTO Orbitals. Cluster 1: Particle ACN (a) – GAS (b), Hole ACN (c) – GAS (d). Cluster 2: Particle ACN (e) – GAS (f), Hole ACN (g) – GAS (h). Cluster 3: Particle ACN (i) – GAS (j), Hole ACN (k) – GAS (l). Iso-values=0.020000.

|  |  |
| --- | --- |
| (a) | (b) |
|  |  |
| (c) | (d) |
|  |  |
| (e) | (f) |
|  |  |
| (g) | (h) |
|  |  |
| (i) | (j) |
|  |  |
| (k) | (l) |

Figure S14 – NPX NTO Orbitals. Cluster 1: Particle ETH (a) – GAS (b), Hole ETH (c) – GAS (d). Cluster 2: Particle ETH (e) – GAS (f), Hole ETH (g) – GAS (h). Cluster 3: Particle ETH (i) – GAS (j), Hole ETH (k) – GAS (l). Iso-values=0.020000.

|  |  |
| --- | --- |
| (a) | (b) |
|  |  |
| (c) | (d) |
|  |  |
| (e) | (f) |
|  |  |
| (g) | (h) |
|  |  |
| (i) | (j) |
|  |  |
| (k) | (l) |

Figure S15 – NPX NTO Orbitals. Cluster 1: Particle WAT (a) – GAS (b), Hole WAT (c) – GAS (d). Cluster 2: Particle WAT (e) – GAS (f), Hole WAT (g) – GAS (h). Cluster 3: Particle WAT (i) – GAS (j), Hole WAT (k) – GAS (l). Iso-values=0.020000.

| (a) | (b) |
| --- | --- |
|  |  |
| (c) | (d) |
|  |  |
| (e) | (f) |
|  |  |
| (g) | (h) |
|  |  |
| (i) | (j) |
|  |  |
| (k) | (l) |

Figure S16 – NPX^-^ NTO Orbitals. Cluster 1: Particle ACN (a) – GAS (b), Hole ACN (c) – GAS (d). Cluster 2: Particle ACN (e) – GAS (f), Hole ACN (g) – GAS (h). Cluster 3: Particle ACN (i) – GAS (j), Hole ACN (k) – GAS (l). Iso-values=0.020000.

| (a) | (b) |
| --- | --- |
|  |  |
| (c) | (d) |
|  |  |
| (e) | (f) |
|  |  |
| (g) | (h) |
|  |  |
| (i) | (j) |
|  |  |
| (k) | (l) |

Figure S17 – NPX^-^ NTO Orbitals. Cluster 1: Particle ETH (a) – GAS (b), Hole ETH (c) – GAS (d). Cluster 2: Particle ETH (e) – GAS (f), Hole ETH (g) – GAS (h). Cluster 3: Particle ETH (i) – GAS (j), Hole ETH (k) – GAS (l). Iso-values=0.020000.

| (a) | (b) |
| --- | --- |
|  |  |
| (c) | (d) |
|  |  |
| (e) | (f) |
|  |  |
| (g) | (h) |
|  |  |
| (i) | (j) |
|  |  |
| (k) | (l) |

Figure S18 – NPX^-^ NTO Orbitals. Cluster 1: Particle WAT (a) – GAS (b), Hole WAT (c) – GAS (d). Cluster 2: Particle WAT (e) – GAS (f), Hole WAT (g) – GAS (h). Cluster 3: Particle WAT (i) – GAS (j), Hole WAT (k) – GAS (l). Iso-values=0.020000.
